# A model argument for accurate EF

**DOI:** 10.1186/1532-429X-18-S1-T12

**Published:** 2016-01-27

**Authors:** Geetha Rayarao, Mark Doyle, Victor Farah, Diane V Thompson, June A Yamrozik, Ronald Williams, Moneal Shah, Robert W Biederman

**Affiliations:** grid.413621.30000000404551168Cardiac MRI, Allegheny General Hospital, Pittsburgh, PA USA

## Background

CMR still suffers from several sources of inaccuracy in measuring LV volumes and EF. Typical standard deviations (SD) between readers for EF range from 4% - 7.5% (Quantification of LV function: Suinesiaputra A, et al. J Cardiovasc Magn Reson. 2015 Jul 28;17(1):63). Further, since the data range of agreement for 95% of data is 4SD (i.e. 16% - 30%), it is common practice to adjust EF based on a visual assessment. This presents several major problems in that there is no guarantee that visual EF is a good guide and that adjustments to EDV and ESV will result in the SV being adjusted correctly. Here we present a technique termed ‘Removing Endocardial Measured Overage Directionally using External Leverage' (REMODEL) that accomplishes intuitive simultaneous corrections of EF and SV.

## Methods

For a group of patients (N = 107) we applied phase velocity mapping (PVM) to the aorta to calculate SV. Using previously described aortic coupling conditions, we also calculated EF from the aortic PVM data. These measures were used to estimate EDVest and ESVest using the following simultaneous equations:

EDVest = SV/EF

ESVest = (SV/EF) - SV

where SV and EF are derived from the aortic PVM data.

**Figure 1:**
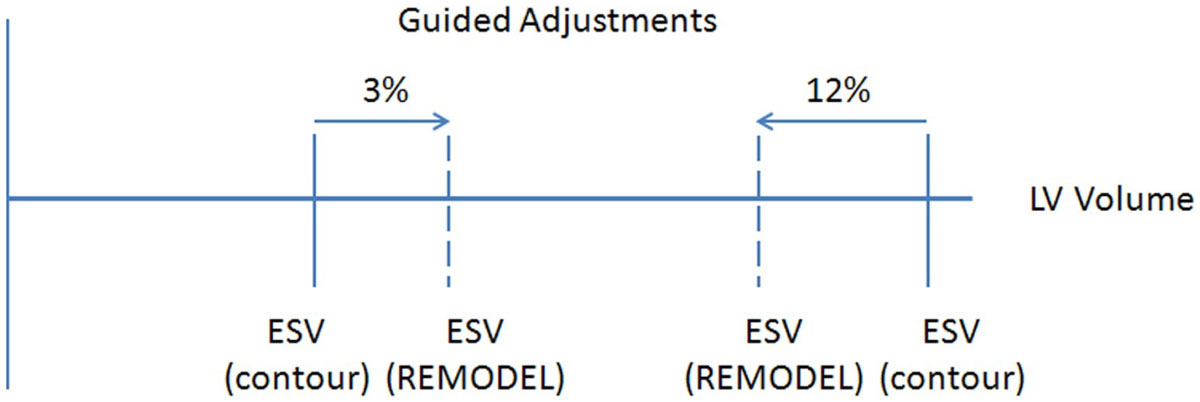
**Example of REMODEL adjustments that direct ESV and EDV separately**.

These aortic estimates of EDVest and ESVest provide the magnitude and direction of adjustment to apply to the volumetric contours encompassing EDV and ESV (Figure 1). Here, to demonstrate this proof of concept, we manually adjusted the volumetric EF by 1SD if it was beyond 1SD of agreement with the aortic calculation.

## Results

**Figure 2:**
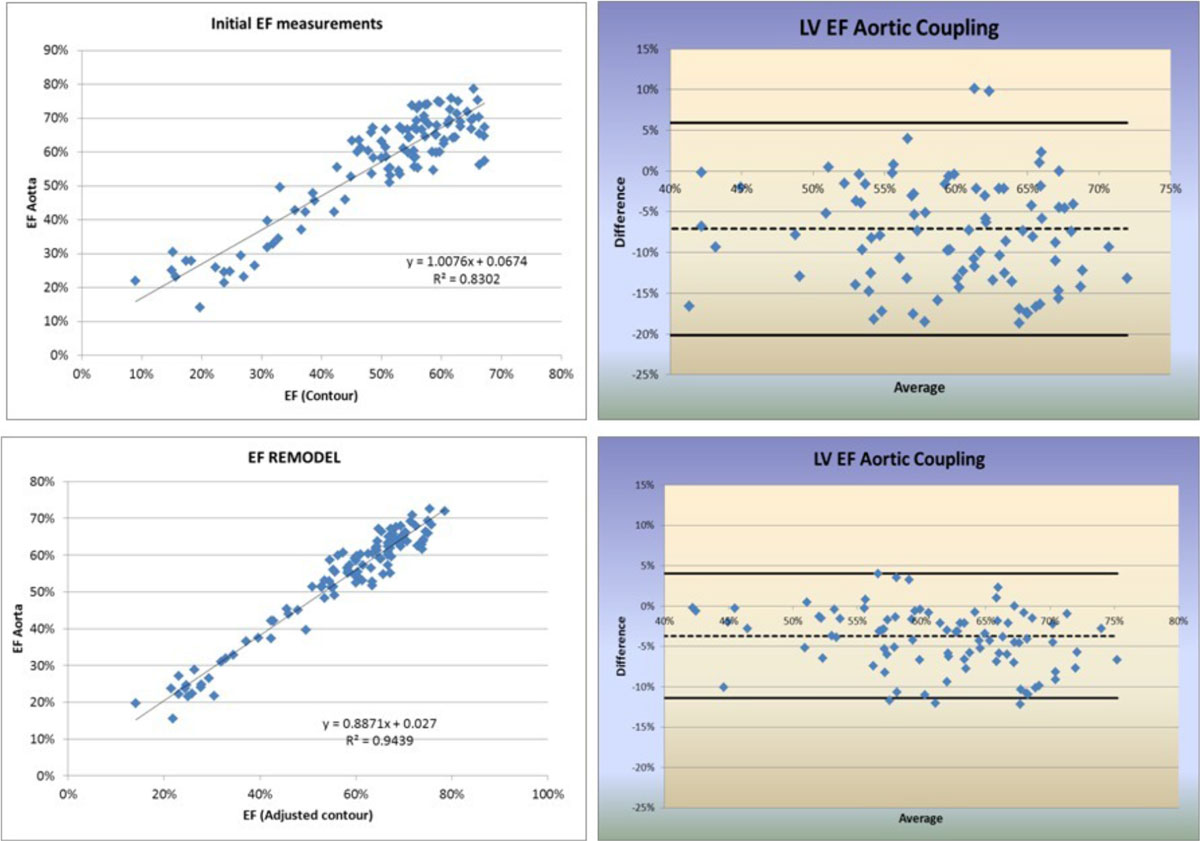
**Correlation and Bland-Altman plots for initial and REMODELED data**.

The contour-determined EFs ranged from 9% to 67%, with corresponding EDV's ranging from 57 ml to 525 ml and ESVs ranging from 21 ml to 408 ml. The aortic-calculated EF correlated well with the contour-measured EF (r = 0.91) with an average difference of 7.1% and a SD of 6.5% (Figure 2). Adjusting the contour-measured EF brought it into closer agreement with the aortic-calculated EF (r= 0.96) importantly reducing the standard deviation by 42% to 3.8%. On average, for the cohort, this decreased the LVEF by 3.5% (p < 0.001).

## Conclusions

By using PVM for aortic calculation of SV and EF, it is possible to re-evaluate EDV and ESV making adjustments to LV contours in a systematic manner that simultaneously brings EF and SV into internal agreement between aortic and LV measures. This approach, which only requires a single additional PVM data set, has immediate clinical implication further improving accuracy and reproducibility of EF.

